# Factors influencing N95 respirator use among healthcare workers in a TB specialized hospital, Bangladesh

**DOI:** 10.1371/journal.pone.0331726

**Published:** 2026-05-18

**Authors:** Sayeeda Tarannum, Abu-Hena Mostofa Kamal, Mohammad Tauhidul Islam, Md. Ariful Islam, Kamal Ibne Amin Chowdhury, Shirina Akhter, Afsana Sharmin, Emily S. Gurley, Sayera Banu, Md. Saiful Islam

**Affiliations:** 1 Department of Global Public Health, Karolinska Institute, Stockholm, Sweden; 2 Program for Emerging Infections, Infectious Disease Division, International Centre for Diarrhoeal Disease Research, Dhaka, Bangladesh; 3 Department of Humanities and Business (HUM), Khulna University of Engineering and Technology, Khulna, Bangladesh; 4 Johns Hopkins Bloomberg School of Public Health, Baltimore, United States of America; 5 School of Population Health, University of New South Wales, Kensington, Australia; Addis Ababa University, College of Health Sciences, ETHIOPIA

## Abstract

**Background:**

Healthcare workers (HCWs) in public tertiary care hospitals, including chest diseases hospitals, experience a substantial tuberculosis (TB) burden, with 40−48% demonstrating baseline TB infection and incidence rates of 4.2–4.8 per 100 person-years, indicating occupational exposure in high TB-burden settings. TB infection prevention and control (IPC) guidelines recommend that HCWs wear N95 respirators in pulmonary TB patient areas. To ensure appropriate use of N95 respirators by HCWs, an understanding of the barriers and facilitators associated with respiratory protection is needed. This study explored factors influencing N95 respirator use and reuse among HCWs in a TB-specialized tertiary care hospital in Bangladesh.

**Methods:**

Between December 1, 2013, and June 05, 2014, a field team consisting of five social scientists conducted a qualitative study to explore the factors influencing N95 respirator use and reuse among HCWs in a TB-specialized, tertiary care hospital in Bangladesh. We initially conducted a day long training workshop with 28 HCWs (doctors, nurses and ancillary workers) working in two multi-drug resistant (MDR) TB inpatient wards on the proper use, reuse, and storage of respirators. Participants also underwent N95 respirator fit testing and received a two-month supply (60 days) of N95 respirators. We also conducted observations with 22 HCWs before and after the workshop to document use of N95 respirators and in-depth interviews with the 24 HCWs to explore TB risk perceptions, and the reported barriers and facilitators of N95 respirator uses.

**Results:**

At baseline, N95 respirators use was high among nurses (8/9), whereas none of the doctors and ancillary workers used N95 respirators. Post-workshop observation findings showed that the use of N95 respirators remained highest among nurses (11/12) compared to doctors (1/5) and ancillary workers (2/5). Perception that N95 prevents germs from entering the body, the feeling of increased confidence when wearing them and concerns about MDR-TB were identified as key motivators for respirator use. In contrast, misperceptions that long-term exposure to TB confer immunity against infection, discomfort, lack of motivation from senior colleagues, pain, difficulties in accessing respirators, communication challenges, fogging of spectacles and cumbersome storage for reuse were identified as the main barriers to consistent N95 respirator use by HCWs.

**Conclusion:**

N95 respirator use among HCWs was feasible but varied substantially across professional groups, with consistently higher uptake among nurses. Persistent behavioral, structural, and institutional barriers limited consistent use. Addressing misconceptions, improving access and storage systems, strengthening leadership support, and reinforcing institutional policies may enhance adherence to respirator use in high TB-burden settings.

## Introduction

Tuberculosis (TB) remains the leading cause of death from a single infectious agent worldwide and ranks among the top 10 causes of death globally, with an estimated 10.7 million people developing TB and 1.23 million deaths reported in 2024 [1]. The low- and middle-income countries (LMICs) have a high tuberculosis (TB) burden [2]. TB is caused by *Mycobacterium tuberculosis bacilli*. Transmission occurs when individuals with active pulmonary or laryngeal TB expel infectious droplet *nuclei* through coughing, sneezing, speaking, shouting, laughing, or singing [3]. These airborne particles, typically 1–5 µm in diameter, can remain suspended in the air for extended period and disperse throughout a room or building [4]. When inhaled by susceptible individuals, they can reach the distal airways, leading to infection [5,6]. Respiratory protective equipment, such as masks and respirators, plays a critical role in preventing airborne transmission. Healthcare workers (HCWs) are at particularly high risk of acquiring TB infection due to repeated and prolonged exposure to TB patients in healthcare settings where infection prevention and control (IPC) measures are inadequately implemented [7]. In Bangladeshi public tertiary care hospitals, including chest diseases hospitals, HCWs experience a substantial TB burden, with 40–48% demonstrating baseline TB infection and incidence rates of 4.2–4.8 per 100 person-years, indicating continues occupational exposure in high TB-burden settings [7]. To protect HCWs from occupational TB infection, the World Health Organization (WHO), US Centers for Disease Control and Prevention (CDC), and Bangladesh National TB Control Program (NTP) recommend the use of N95 respirators among HCWs as part of a multi-component strategy to prevent transmissions of *M. tuberculosis* in health care settings [8–10].

Published literature shows that N95 respirators provide strong protective efficacy for HCWs against TB, offering a tight facial seal and superior filtration efficiency, whereas cloth or surgical masks block less than half of aerosol particles, leaving HCWs highly vulnerable to inhaling mycobacterium tuberculosis [11–14]. N95 respirator can maintain adequate protective performance against TB even after being reused for up to three continuous days [12]. Given N95 respirator’s high cost, respirators reuse is a common practice in some LMICs, where the same respirator is worn multiple times after being safely stored between uses [3,4].The CDC and WHO have both issued guidance on the safe reuse or extended use (wearing a respirator continuously for an extended period without removal between patient encounters) of respirators by HCWs in instances where respirators are in limited supply [15].

In prior studies, concerns have been raised regarding the packaging and storage of reused respirators, the need for clear and accurate labelling, the distortion or loss of labels during handling, and the potential for contamination before reuse [16]. Moreover, heat, breathability, tightness, ease of speaking and tolerance during extended use, itchiness, ease of displacement and ear discomfort also influence the usability of respirators [17]. To ensure proper respirator use among HCWs in Bangladesh, a comprehensive understanding of individual level factors (e.g., risk perception and knowledge), administrative systems (e.g., supply chain, storage protocols, supervision), and cultural dynamics (e.g., hierarchical norm and peer influence) is essential. In this study, we therefore explored the individual, administrative and cultural factors influencing N95 respirator use among HCWs in a TB-specialized, tertiary care hospital in Bangladesh.

## Materials and methods

### Study design, site, and participants

Between December 1, 2013 and June 05, 2014, we conducted this exploratory qualitative study at the National Institute of Disease of the Chest and Hospitals (NIDCH), a 685-bed tertiary care TB specialized hospital in Dhaka, Bangladesh [18]. The hospital provided services to approximately 100,000 TB and respiratory patients per year, referred from hospitals and specialized doctors throughout the country [12]. Twenty-eight doctors, nurses, and ancillary workers (cleaners, ward boys, ayas) who worked on the hospital’s two multi-drug-resistant (MDR) TB wards were invited to participate. We selected all HCWs purposively, who worked in the MDR-TB wards, having TB related experiences and their roles in TB prevention.

### Data collection

Between December 1, 2013, and June 05, 2014, a field team consisting of five social scientists conducted a qualitative study to explore the factors influencing N95 respirator use and reuse among HCWs in a TB-specialized, tertiary care hospital in Bangladesh. The team talked to the hospital director and the heads of the study wards to describe the study objectives and obtain permission to conduct the study. After receiving permission, they conducted the study in two MDR-TB inpatient wards. These wards were selected based on hospital stakeholders’ recommendations and their high patient load of MDR-TB cases, ensuring relevance to the study objectives. Ongoing research activities within these wards further supported efficient implementation within the available budget and timeline. The team then conducted a training workshop on respirator use and fit testing which was attended by doctors, nurses and ancillary workers, including ayas (female attendants), word boys and cleaners. The biosafety and biosecurity team from the International Centre for Diarrhoeal Disease Research, Bangladesh (icddr,b) led the workshop and provided the training. The team described TB transmission pathways, the role of N95 respirators in preventing TB and other respiratory diseases. The experts facilitated hands-on training on proper use, reuse, and storage of respirators, during which participating HCWs also underwent qualitative fit testing and received appropriately sized respirators. The fit testing involved instructing participants on correct respirator donning, exposing them to a test agent (Bitrex/saccharin) while performing standardized breathing and movement exercises, and confirming an adequate seal when no taste or odor was detected [19]. During the training workshop, HCWs were provided with N95 respirators and instructed on appropriate storage practice between uses. They were advised to allow used respirators to air-dry completely before storage and to keep them in a clean, resealable plastic (zip-lock) bag to prevent contamination. To maintain ventilation, two small holes were made in each bag using a punch machine. Participants were also instructed to fold the outer surface of the respirator inward to protect the inner surface, label their storage bag with their name to prevent accidental use by others, and dispose of the respirator if it became damp, soiled, or damaged. We provide a total of 171 National Institute for Occupational Safety and Health (NIOSH) certified, N95 respirators (3M Corporate Headquarters 3M Center St. Paul, MN 55144−1000, USA) to 24 HCWs (mean 6.6 respirators/HCWs) for 62 days. We maintained a register to document the date and time that respirators were provided. We tracked use time of respirators and instructed the HCWs to use a new respirator after eight hours of total use during the care of TB patients. If HCWs reported feeling uncomfortable reusing a respirator, they were allowed to take a new one. We also instructed them to discard used respirators in the designated waste receptacles located outside the wards.

A total of 28 HCWs attended the workshop, 24 in in-depth interviews. Additionally, we observed 22 HCWs. We conducted 84 hours of observation: 24 hours before the workshop and 60 hours after the workshop to record use of N95 respirators followed by 24 semi-structured, in-depth interviews (IDIs) with 10 doctors, 8 nurses and 6 ancillary workers to understand the barriers and motivators for N95 respirator uses. Same method was followed during pre-and post-workshop observation. Recognizing that HCWs activities vary by shift, the team conducted 3 hours structured observation sessions across daytime, evening, and night periods to capture variation in exposures and N95 respirator use. Each observer focused on a maximum of 10 patients. Observers positioned themselves unobtrusively within the ward, wore N95 respirators during observation, and recorded practices using detailed handwritten field notes. All the participants were native Bengali speakers, so the interviews were conducted in Bangla. We asked the respondents about the motivating factors, such as safety concerns and professional obligations, and demotivating factors, including technical issues, physiological barriers, risks of contamination, and socio-cultural barriers for using N95 respirators. We conducted 24 interviews and recorded them using a digital audio recorder. The average duration of the interviews was 42 minutes. The interviews were conducted at the workplace of the respondents suggested by the respondents. We also took detailed notes during interviews.

### Data analysis

A descriptive analysis was performed on the data obtained through the interviews. The research team transcribed the audio recordings verbatim in *Bengali* and then translated the content into English using Microsoft Word. The recordings were reviewed multiple times to ensure the accuracy of the transcriptions. Initially, two researchers examined the field notes to develop a preliminary codebook. The coding framework was informed by the study’s objectives, relevant literature, and both predetermined and emerging themes related to the motivations and challenges of N95 respirator use among HCWs. The transcribed content and field notes were imported into ATLAS.ti version 6.0 to facilitate coding and analysis. The lead researcher reviewed and coded relevant excerpts based on the established codebook. When new data did not fit the existing codes, new codes were developed and incorporated, and this iterative process continued throughout the analysis. Coders held regular discussions to refine and ensure consistency in code definitions and categorization of emerging themes. The analysis was guided by the Health Belief Model (HBM), which includes five key dimensions: (a) perceived susceptibility; (b) perceived severity; (c) perceived benefits; (d) perceived barriers; and (e) cues to action [20].The coded data were organized under broader themes aligned with these HBM dimensions, and thematic summaries were developed in English. All members of the research team collaboratively reviewed the themes and sub-themes to reach consensus and ensure consistency.

### Ethical consideration

We obtained informed, written consent from the hospital authorities for conducting the research activities. We obtained informed written consent from all IDI participants. The study protocol PR#12067 was reviewed and approved by icddr,b’s Institutional Review Board consisting of a Research Review Committee and an Ethical Review Committee.

## Results

A total of 28 HCWs attended the workshop: 12 doctors, nine nurses, and seven ancillary workers. The mean age of the study participants was 40 years. Thirteen (54%) were female, nine (38%) were nurses, 10 (41%) were doctors, and five (21%) were ancillary staff. The average time working in a TB hospital ranged from 4.5 years for doctors to 16.5 years for ancillary workers. As a note, based on the fit testing, icddr,b’s expert found that 11 HCWs required medium and the rest of the HCWs required small-size N95 respirators.

Of the 28 healthcare staff working on the MDR-TB wards, all were invited to participate; however, we excluded one male doctor and one male nurse from fit-testing as both had beards, and a bearded person cannot achieve a full fit test. Another doctor and one ancillary worker were transferred to another department of the hospital and, therefore, excluded from the fit testing and interviews.

### Perceived risk of TB infection

Of 24 interview participants, 18 (75%) perceived that they were at risk of TB infection due to close contact and everyday exposure to TB patients in the wards. Moreover, 7 (29%) participants mentioned that they were afraid of being infected with MDR- TB while providing care for patients. One nurse stated,

*I will get TB if I talk to them. I will inhale their breath; they will inhale mine; All of us working in this hospital are at risk* of TB infection*. Staff, patients, and visitors are all at risk*.

One ancillary worker added that she was aware of the risk of being infected with TB, but she was not afraid, because she believed that “one gets sick from God’s will”. Another nurse believed that protection against TB was entirely up to God.

On the other hand, one ancillary worker mentioned that he was not at risk of TB because he maintained cleanliness, washed his clothes regularly, and took a bath after returning home every day. A quarter of participants (6/24) reported that they were concerned about getting TB infection during their initial days after joining the TB hospital. Over time, they became accustomed to working with TB patients, and their concern about TB infection diminished.

### Perceived susceptibility

One nurse and one doctor said that they heard from a senior colleague that all HCWs aged 40 and older are likely to have latent TB infection. Another doctor quoted from one of his senior colleagues that none of the doctors who worked at NIDCH were infected with TB, so he should not be worried about acquiring TB infection. Three participants provided an example that they observed very few HCWs working in the hospital developing active TB, so they felt confident in the low probability of being infected with TB. One doctor stated,

*Our senior colleagues encourage us by saying they have been serving at this hospital for many years, and they have not been infected. So, we will not be affected. We have been working here with this belief that we would not be infected.*

### Perceived need for N95 respirators

Three doctors reported that they should use a respirator in the MDR-TB ward, the TB ward, and while examining presumptive TB patients at outpatient clinics. Additionally, four doctors stated that the use of the respirator was needed if someone remains in close contact with any TB patient, especially in MDR-TB ward. One doctor stated,

*The highest advantage is feeling safe. N95 respirator provides maximum protection against TB infection. I am feeling safe because I am wearing a respirator that will protect me from TB infection. Moreover, it gives me mental satisfaction and confidence since I am using respirators, I will not be infected.*

### Use of N95 respirators

During the 20 hours of observation conducted before the workshop, 16 HCWs were observed in the two MDR-TB wards: nine nurses, six ancillary workers, and one doctor. Eight of the nine nurses were observed wearing N95 respirators, whereas none of the ancillary workers or the doctor used an N95 respirator (Table 1).

**Table 1:** Use of N95 respirators among HCWs at baseline and after the training workshop in a tertiary care hospital, Bangladesh-2014.

Pre-workshop observation (20 hours)						Post-workshop observation (64 hours)				
Profession	Total	N95	Cloth mask	No mask	Exposure (minute)	Total	N95	Cloth mask	No mask	Exposure (minute)
Nurse	9	8	0	1	226	12	11	0	1	208
Ancillary worker	6	0	1	5	294	5	2	1	2	100
Physician	1	0	0	1	98	5	1	0	0	114

All interview participants reported that they used the N95 respirators provided by the icddr,b team. However, during the 64 hours of observation conducted after the workshop, 22 HCWs were observed in the two MDR-TB wards: 12 nurses, five ancillary workers, and five doctors. Eleven of the 12 nurses, two of the five ancillary workers and one of the five doctors were observed wearing N95 respirators (Table 1).

Participants reported that wearing N95 respirators when in contact with TB patients (n = 7), before entering the MDR-TB ward (n = 8), or general TB ward (n = 4), while serving food (n = 2), assisting patients in wheelchairs for examinations (n = 3), and during emergency situations (n = 1).

### Factors influencing the use of N95 respirators

#### Perceived benefit of using N95 respirators.

Sixty percent (16/24) of the participants asserted that germs cannot enter through an N95 respirator; therefore, it can protect them from TB infection. Ten (42%) reported that they felt protected, safe, confident, and secure when they used an N95 respirator (Table 2). One doctor mentioned that an N95 respirator could protect against the TB germ if it is used appropriately. He said,

**Table 2:** Factors influencing N95 respirator use among HCWs Working on MDR-TB Wards in a public, tertiary-care hospital, Bangladesh, 2014.

Category	Motivators	N	Barriers/ Demotivators	N
**Perceived benefits**	Perception that germs cannot penetrate an N95 respirators	16		
	Feeling protected, safe, mentally satisfied, confident and secured	10		
	Using before close contact with TB patient, MDR-TB ward, conducting bronchoscope procedure or examining a presumptive TB patient provide protection	20		
**Perceived barrier**			Physical discomfort (pain from nose, itching, difficulty in breathing)	11
			Blurred vision due to fogging of spectacles	6
			Difficulty communicating or counselling patients while wearing respirators	5
			Incompatibility with nursing hats	6
			Seasonal discomfort (Heat, humidity)	6
			Lack of knowledge on reuse duration, storage, and contamination risk	19
			Limited supply and inadequate storage materials	15
			Feeling uncomfortable wearing respirators when senior colleagues do not	9
			Beliefs that diseases are God’s will	2
			High cost of N95 respirators	4
**Cues to action**	Awareness of MDR-TB risk and clinical procedures such as bronchoscopy serv as reminders to wear respirators	2

*…the highest advantage is feeling safe. N95 respirator provides maximum protection against TB infection. I am feeling safe because I am wearing a respirator that will protect me from TB infection. Moreover, it gives me mental satisfaction and confidence since I am using respirators, I will not be infected.*

#### Perceived barriers.

Seventy percent of the doctors (7/10) said that they preferred inhaling fresh air to wearing a respirator. Furthermore, most of the doctors (6/10) perceived that the N95 respirator could ensure 95% protection against TB germs which denotes there always remain 5% chance of being infected despite wearing a respirator. They also perceived that contaminated air could easily pass through the respirator if there is any face-seal leakage. One of the doctors mentioned,

*The respirator does not stay in the same position as when I first put it on (on face); it becomes loose and shifts. I feel uncomfortable when it is displaced. I worry that germs may enter my respiratory tract due to loose fitting and displacement.*

#### Concern about N95 respirator storage and re-use.

Lack of knowledge on reuse duration, storage and contamination risk were mentioned as perceived barriers (Table 2). A few participants (3/24) believed that an N95 respirator can become a source of TB infection if it is being reused and stored in the same zip-lock bag several times. One third of the doctors (3/10) added that carrying respirators either in a bag or inside their pocket was not safe or feasible. They added that respirators might get contaminated with other microorganisms in the zip-lock bag (Table 2). They also reported that they did not have any idea about how long a respirator could be stored in the bag and for how long it would remain safe for reuse. One doctor stated,

*I do not want to use a respirator that I have to carry in my hand or pocket. It would have been better if we could store it in a backpack. Keeping it in my pocket or reusing it may increase the chance of infection. I do not like keeping a used respirator in my pocket. If I can’t store the respirator properly, I will not reuse it.*

A few participants (3 out of 24) also reported that their respirators became torn or damaged while being carried in a pocket or stored in bag.

#### Discomfort and difficulties in breathing.

Nine participants (37.5%) reported that they could not wear an N95 respirator for more than an hour continuously, as it caused breathing difficulty. One doctor said that an N95 respirator could be worn for 20–30 minutes at a time. The doctors reported feeling suffocation and breathing difficulty when using a respirator for an extended period. One ancillary worker stated that she wore respirators while transporting patients but could not continue after transporting the patient in the ward due to feelings of suffocation. All the nurses (8/8) reported that it was not possible to wear a respirator throughout their entire duty hours (8 hours); they only used the respirator while providing patient care in the ward. One doctor said she felt itchy when she used the respirator. One nurse noted discomfort and pain over the nasal area when she used the respirators. Ancillary workers also reported similar experiences. One ancillary worker reported,

*I was accompanying a patient on a trolley while wearing a respirator. I started feeling suffocated and removed it. I often feel suffocated while I wear a respirator and that’s why I do not use them*.

#### Climatic conditions.

One quarter of participants (6/24) reported that during the summer, sweat and retained exhaled air caused additional facial heat and discomfort while wearing respirators (Table 2). All participants reported spending extended periods with patients. On average doctors worked 6.5 hours per day and nurses 8 hours per day, six days per week.

#### Difficulties in carrying a respirator.

Most of the participating doctors (7 out of 10) reported that carrying a respirator from one ward to another was a barrier. Since respirators were not available in the wards and doctors’ offices were located more than one hundred meters away, they had to carry their respirators during ward rounds or while visiting patients. For example, one doctor noted that *the respirator felt like an additional burden during rounds, as I also had to carry student books and patients’ investigation files, X-rays, and pathology reports.*

#### Forgetfulness during emergencies.

Doctors frequently reported forgetting to bring their respirators during ward rounds. One nurse explained that in emergency situations, for example, when a patient’s condition became critical or when a doctor called for urgent assistance -nurses often rushed to the ward and occasionally forgot to wear a respirator. Similarly, one ancillary worker mentioned that she did not use an N95 respirator while serving food to patients, as she needed to move quickly to distribute meals.

One nurse reported *that sometimes in an emergency, for example if the patient’s condition became critical or if the visiting doctor on the ward called for an emergency, then the nurses had to run to the ward and sometimes forgot to wear a respirator.*

#### Socio-cultural norms and institutional factors.

Half of the participating doctors (5/10) informed us that HCWs’ use of a respirator was not common in the hospital. A doctor explained that wearing a gown is a standard precaution for doctors, however, very few HCWs comply with that standard, and similarly non-adherence to respirator use became a hospital norm. He said,

*The culture of self-protection has not been developed yet. For example, doctors should wear aprons, but we do not use them. Non-adherence has become a norm or culture. This problem exists because of a lack of habit.…many of us even make fun of someone who puts on a mask.*

On the other hand, 3/8 nurses reported that there was a limited supply of respirators. They were instructed to use a respirator for seven days, but sometimes they had to use a respirator for two weeks owing to the limited supply. In addition, respirators were too expensive for HCWs to buy on their own. Therefore, they reused the respirators for 3 hours a day for seven days a week or sometimes more than that. One nurse stated,

*We stay with patients in the wards most of the time, taking care of them during ward rounds and giving injections. However, during the evening or night shift, we spend less time with patients, while in the morning shifts, we must stay for longer hours.*

#### Nursing uniforms.

Some nurses (3 out of 8) reported that their uniforms, particularly the traditional white hard hospital hats worn by female nurses posed a barrier to respirator use, as they prevented proper fitting of the N95 respirator. One nurse-in-charge mentioned that nurses faced difficulties both putting on and removing respirators because of the hats. To address this issue, they replaced hats with caps with cloth head coverings while working in the MDR-TB ward, where frequent patient contact was required.

One nurse explained,

*We are used to wearing a nursing hat as part of our uniform, and it makes us look beautiful. We had difficulties wearing an N95 respirator because of this large and specially designed hard materials. Now, we wear a small cloth cap so we can wear a respirator without any issues. See how much we are sacrificing to wear an N95 respirator.*

### Limited motivation from senior colleagues

Over half of the participating doctors (6/10) mentioned that they followed the example of their senior colleagues regarding respirator use (Table 2). They hesitated, felt awkward, and remained confused about the necessity of N95 respirators because their senior colleagues did not wear them. One doctor said,

*The main problem is that when you observe that your senior colleague is not using a respirator, you cannot use it. Suppose my professor is not using a respirator when he is visiting patients in the ward. In that case, how can I wear a respirator as a junior doctor?*

#### Difficulties in counselling and communicating with patients.

Five of the study participants (5/24) reported that the use of a respirator was a barrier when communicating with patients. One doctor stated that she had to speak loudly while wearing a respirator. It also hampered their expressions and natural flow of speaking, she added. One nurse reported that the facility was an academic institute, and they had to communicate frequently with professors regarding patients’ conditions; the use of respirators became a barrier as it hindered the natural flow of conversation; therefore, they could not continue using a respirator (Table 2). Six HCWs noted that respirators use caused their spectacles to fog, resulting in blurred vision.

### Cues to action

Nearly half of the participants (10/24) mentioned that they were concerned about MDR-TB. The MDR-TB ward itself served as a reminder for HCWs to wear respirators before entering. One doctor explained that TB patients exhale large number of bacteria during bronchoscopy, as the procedure induces significant coughing.; this, he noted, reminded him to wear an N95 respirator. One nurse added,

*All hospital staff who deal with TB patients emphasized wearing respirators. Everyone keeps in mind that they need to wear a mask before entering inpatient wards. Whenever we go to the inpatient ward or decide to go, we wear the respirator.*

## Discussion

This study found that N95 respirator use and reuse among HCWs was feasible when clear instructions on reuse and storage were provided, along with designated storage areas and adequate resources. However, patterns of use and reuse varied across HCW groups and were influenced by multiple challenges These include administrative barriers, physiological discomfort, concerns about contamination prior to reuse, supply constraints, a lack of role models and discouragement from senior staff. Additional socio-cultural and technical barriers such as need to carry respirators between wards, forgetting to bring them from duty stations and difficulties communicating with patients while wearing them-also hindered consistent use. Our findings are consistent with previous studies showing that factors such as risk perception, perceived disease severity, motivation, equipment availability and accessibility, and cultural influences, including values, behavior, encouragement, and peer feedback affect N95 respirator use [21–25].

The high acceptance and consistent use of N95 respirators among nurses, together with the observed post-workshop improvement in use among doctors and ancillary workers, suggest that the identified challenges and barriers are amendable to change. In our study, nurses were found to be more consistent and frequent users of N95 respirators compared with doctors and ancillary staff. This may be attributed to their longer and more frequent exposure to TB patients, greater perceived risk, and easier access to respirators, which were stored near their duty stations adjacent to the study wards [26].

We identified persistent supply constraints in the study hospitals. In MDR-TB inpatient ward, N95 respirator practices should be embedded within formal IPC frameworks that priorities extended use of N95 respirators when caring for cohorts of patients as this minimizes repeated handling and reduces the risk of contamination [27]. Where limited reuse unavoidable due to sustained shortages and high cost, IPC protocols should require clear individual labelling to avoid cross use, immediate disposal of respirators that are visibly contaminated, damaged, or difficult to breathe through [27].

Overall, our findings suggest that simply providing N95 respirators may not be sufficient to ensure consistent use. A comprehensive strategy that includes education on TB transmission pathways, the role and proper use of N95 respirators, fit testing, and addressing logistical, behavioral, and cultural barriersis essential for enhancing adherence to respiratory protection practices among HCWs [26]. Training on personal protective equipment has been shown to enhance HCWs’ risk perception and adherence [28]. In our study, although the perceived need for N95 respirator use was low, particularly among doctors and ancillary workers, training on TB transmission and respirators use improved their knowledge of TB risk, respirator use, reuse and storage. It also enhanced their understanding of the role of respirators in TB prevention, which is likely to improve adherence if other barriers are addressed. Similarly, implementation of infection control policies and evidenced based training on correct use, fit testing, and storage has been shown to improve HCWs’ compliance with personal protective equipment, including N95 respirators [29,30].

Discomfort associated with respirator use was an issue impacting the acceptability. Our study also identified that a few of the HCWs were not adherent to respirator use due to breathing difficulties which was consistent with prior studies that reported discomfort associated with respirator use affected the adherence among HCWs in the in-patient wards in tropical countries [31,32]. In hot humid temperatures, use of N95 respirators may increase concentration of CO^2^ and reduced concentration of O^2^ for inhalation and cause dizziness, nausea and headache [32,33]. HCWs felt comfortable wearing N95 respirators in summer with a temperature between 20 and 24 degrees Celsius and a relative humidity between 76%− 96% [24,34]. It might be discomfortable to use respirators, particularly during the monsoon in Bangladesh where the average annual temperature is found to be 24.7 °C and 34.6 °C [35].

During emergencies, respirator use among HCWs was low due to their heavy involvement in managing critically ill patients and forgetfulness to take a respirator with them. N95 respirators were stored in doctors’ chambers or lounges located some distance from the study ward, which posed a significant barrier to comply with the recommendations of N95 respirator use before entering MDR TB wards. A study revealed that adherence is enhanced when face masks and respirators were readily available [35,36]. Therefore, ensuring convenient storage of respirators near patients’ wards such as designated PPE storage areas managed by hospital authorities could increase consistent respirator use among doctors.

The use of N95 respirators is key component of airborne IPC and should be implemented in combination with administrative and environmental controls. A study conducted in the United Kingdom invented a technique named ‘*singh thatta’* technique [37]. It is an under-mask plastic made bearded cover for those who have beards that cannot shave for religious or other reasons, allowing them to wear a tight-fitting mask (like an N95). This technique has proven successful in achieving fit testing of the respirators.

Our study findings suggest that many of the barriers to HCWs respirator use could be addressed by maintaining adequate respirator supply, conducting routine respirator fit testing, and regular training on N95 respirator use [38]. In addition, monitoring and audit can increase the use of N95 respirator use. Nanomaterial based respirators can significantly increase filtration efficiency, breathing comfort, and antibacterial/antiviral properties of the respirators and thereby improving user adherence [39]. Moreover, provision of respirators with an exhalation valve may help to address discomfort due to difficulty breathing and warm temperatures [40]. Posters can be hung in the doctor’s room near the wards entrance as a cue to action to improve adherence [41]. Additionally, having senior doctors who function as role models for junior doctors, and physician IPC champions may also increase adherence among HCWs [42].

Our study has limitations. First, as this study was conducted in a single specialized hospital in Bangladesh, the findings may not be generalizable to other healthcare settings, including general tertiary care hospitals. Second, the study relied on self-reported information and thus is subject to reporting bias. Finally, the compliance rate might vary during winter due to low temperature and less discomfort from sweating inside the respirator [43]. Nevertheless, the barriers and facilitators identified in this study are aligned with other recent studies conducted in TB specialized hospitals and general public tertiary care hospitals [44,45]

## Conclusion

This study found that N95 respirator use among HCWs was feasible but varied across professional groups. Key barriers included accessibility issues, forgetfulness, discomfort, communication difficulties, interference from nursing hats, limited knowledge about reuse and storage, and non-adherence among senior physicians. To improve adherence, context-specific strategies are needed-such as strengthening TB IPC systems, ensuring regular supply of N95 respirators and storage materials, providing ongoing training, fostering motivation and role modelling by senior clinicians. Ongoing monitoring and audits are necessary to ensure proper compliance with N95 respirator use among healthcare workers in Bangladesh. Establishing a multidisciplinary TB IPC committee comprising doctors, nurses, and ancillary workers could support these efforts through effective supervision, regular refresher training, and the promotion of a workplace culture that prioritizes respiratory protection.

## Supporting information

S1 File Interview Guideline. (DOCX)

S2 File Global Inclusivity Research. (DOCX)
