# Supplementary material for: Factors influencing N95 respirator use among healthcare workers in a TB specialized hospital, Bangladesh
Source: PLoS One. 2026 May 18;21(5):e0331726. doi: 10.1371/journal.pone.0331726 (PMC13183184; doi:10.1371/journal.pone.0331726)
Supplement: S1 File — (DOCX) [file pone.0331726.s001.docx]

**Supplementary 1: Interview guidelines for doctors, nurses and support workers**

Age, sex, education, work status, supervisory status

How often do you deal with TB patient?

What protective instruments were available (before the intervention) in your facility? Did you use them? If yes, how often? If no, why?

Did you use any facial protection? How would you compare N95 mask with the previously used facial mask?

When a TB patient is admitted, what steps do you take to protect yourself?

What steps do you take for TB patient management?

Do you have concerns treating or working with TB patients?

Did you ever participate in any TB training or workshop? If yes, when and what type of training?

Do you have sufficient access to information about TB care, protective measures and other TB-related subjects to confidently manage patients? If yes, from whom, how?

Do you know the national TB-control guidelines for health care workers? if yes, please explain.

Do you know the policy for dealing with TB patient in your hospital? If yes, please explain?

Do you think you are at risk for TB? If yes, why and how?

In your opinion, which group of caregivers are at highest risk for TB infection?

Which patient area (pediatric or adult) are riskier for getting infected and how?

Do you think TB is a serious disease? If yes, why, if not, why?

Positive consequences of wearing mask?

What are the barriers of wearing mask?

What factors motivate you to wear mask?

What factors de motivates you to wear mask?

Do you feel any discomfort during wearing mask? If yes, what type of discomfort?

Do you think the TB disease might be transmitted from one person to another?

Are there any conflict between job responsibility and wearing mask?

Is there any conflict between nursing dress and wearing masks?

Do you think N95 mask can protect you from being infected?

Do you feel protective during wearing mask?

Do you feel confident of using N95 mask?

What factors remind you to wear mask?

Are you confident how to wear mask?

Do you have any previous experience of using mask?

Do you think TB is a serious disease? Is there any negative impact of TB on life?

Perception on the effectiveness of N95 mask?

What are the critical times for wearing mask and why? How long should we wear mask? And why?

What difficulties do you have while wearing the N95 mask? (For probe: Hot? Heard to breathe through, difficulties to communicate with patients, seniors discouraged wearing mask?)
